# Virtual Reality in Neurosurgery: Beyond Neurosurgical Planning

**DOI:** 10.3390/ijerph19031719

**Published:** 2022-02-02

**Authors:** Rakesh Mishra, M.D. Krishna Narayanan, Giuseppe E. Umana, Nicola Montemurro, Bipin Chaurasia, Harsh Deora

**Affiliations:** 1 Department of Neurosurgery, Institute of Medical Sciences, Banaras Hindu University, Varanasi 221005, India; rakeshmishra_afmc@yahoo.co.in; 2 Department of Neurosurgery, Yenepoya Medical College, Mangalore 574142, India; mdknarayanan@gmail.com; 3 Trauma and Gamma-Knife Center, Department of Neurosurgery, Cannizzaro Hospital, 95100 Catania, Italy; umana.nch@gmail.com; 4 Department of Neurosurgery, Azienda Ospedaliera Universitaria Pisana (AOUP), University of Pisa, 56100 Pisa, Italy; 5 Department of Neurosurgery, Bhawani Hospital, Birgunj 44300, Nepal; troexa@gmail.com; 6 Department of Neurosurgery, National Institute of Mental Health and Neurosciences, Bengaluru 560029, India; harshd@nimhans.ac.in

**Keywords:** augmented reality, virtual reality, mixed reality, neurosurgery, brain tumor, robotic neurosurgery, training, neuronavigation, computed tomography

## Abstract

Background: While several publications have focused on the intuitive role of augmented reality (AR) and virtual reality (VR) in neurosurgical planning, the aim of this review was to explore other avenues, where these technologies have significant utility and applicability. Methods: This review was conducted by searching PubMed, PubMed Central, Google Scholar, the Scopus database, the Web of Science Core Collection database, and the SciELO citation index, from 1989–2021. An example of a search strategy used in PubMed Central is: “Virtual reality” [All Fields] AND (“neurosurgical procedures” [MeSH Terms] OR (“neurosurgical” [All Fields] AND “procedures” [All Fields]) OR “neurosurgical procedures” [All Fields] OR “neurosurgery” [All Fields] OR “neurosurgery” [MeSH Terms]). Using this search strategy, we identified 487 (PubMed), 1097 (PubMed Central), and 275 citations (Web of Science Core Collection database). Results: Articles were found and reviewed showing numerous applications of VR/AR in neurosurgery. These applications included their utility as a supplement and augment for neuronavigation in the fields of diagnosis for complex vascular interventions, spine deformity correction, resident training, procedural practice, pain management, and rehabilitation of neurosurgical patients. These technologies have also shown promise in other area of neurosurgery, such as consent taking, training of ancillary personnel, and improving patient comfort during procedures, as well as a tool for training neurosurgeons in other advancements in the field, such as robotic neurosurgery. Conclusions: We present the first review of the immense possibilities of VR in neurosurgery, beyond merely planning for surgical procedures. The importance of VR and AR, especially in “social distancing” in neurosurgery training, for economically disadvantaged sections, for prevention of medicolegal claims and in pain management and rehabilitation, is promising and warrants further research.

## 1. Introduction

Virtual reality (VR) and augmented reality (AR) have intuitively found utility in neurosurgical planning, considered the increasing availability of papers available on this subject. However, the utility of VR and AR beyond neurosurgical planning remains unexplored and, therefore, opens avenues to expand their applicability. We intend to provide an overview of this vast expanse, beyond mere neurosurgical planning.

VR involves the user being placed in a system that occludes the natural world and generates a virtual world that the user can experience. Depending on whether the virtual world is designed as a virtual environment or convincing substitute for the real world, VR is further classified as non-immersive and immersive, respectively [1]. AR differs from VR in that there is a fusion of the natural and virtual world elements in AR, with superimposition of virtual images (as holograms) over the real-world environment, allowing for the simultaneous perception of (and, therefore, the interaction between) the projected virtual image and natural environment [2,3]. This immersion and interaction were initially achieved by placing the user within active stereoscopic glasses (for immersion) and providing controllers that allowed for interaction and aided immersion with haptic feedback. Modifications to counter the smaller field of view afforded by the glasses, restricted area of work, and absence of head tracking (that allows for perspective change by the user) was made over time [4]. However, these modifications were jarringly unnatural when the virtual image/environment and user’s perspective were misaligned [5,6]. The addition of tracking the user and providing this feedback to the system allowed for a seamless user–environment interface [7]. VR has gradually gained importance in neurosurgery, as illustrated in the article by Tomlinson et al. [7] of VR-based training modules, which enhanced the understanding of neuroanatomy using these systems. Subsequent studies have focused on the utility of VR and AR in neurosurgical planning, drawing disproportionate focus to this one application. We aim to present an updated overview of the concepts, as well as the utilization, implications, and future possibilities of VR in neurosurgery, beyond the bounds of planning alone. Though our present review is not systematic, we have systematically searched and synthesized the review to reach meaningful conclusions. This review focuses on the role of VR in neurosurgery training and simulation, intraoperative utility, neuronavigation, postoperative rehabilitation, pain management, and future challenges.

## 2. Materials and Methods

Though the present review is not intended to be a systematic review of the literature, we systematically conducted our search to remove redundancy and be more comprehensive in our narrative review. Systematic review for VR in neurosurgery, beyond planning, is not feasible, owing to limited homogenous studies. The lack of sufficient studies exemplifies the infancy of this aspect of VR and AR to conduct a systematic review. To construct this narrative review, we searched PubMed, PubMed Central, Google Scholar, the Scopus database, the Web of Science Core Collection database, and the SciELO citation index, from 1989–2021. Two authors (RM and HD) independently reviewed the retrieved studies, with title and abstract for further detailed review. An example of search strategy used in PubMed Central is as: “Virtual reality” [All Fields] AND (“neurosurgical procedures” [MeSH Terms] OR (“neurosurgical” [All Fields] AND “procedures” [All Fields]) OR “neurosurgical procedures” [All Fields] OR “neurosurgery” [All Fields] OR “neurosurgery” [MeSH Terms]). The search was started on 1 November 2020 and finished on 25 August 2021. Title and abstract were screened to identify articles providing discussion on the utility of VR, in addition to neurosurgical planning. Only articles in the English language were included. The full text of the articles was retrieved and reviewed from the eligible list to identify their inclusion to synthesize this review. In addition, the bibliography of the included articles was screened to identify other potentially eligible articles. We have excluded studies on neurosurgery planning and focused on studies exploring the use of VR in other scenarios in neurosurgery.

## 3. Results

Using the search strategy, we identified 487 (PubMed), 1097 (PubMed Central), 275 citations (Web of Science Core Collection database). This review is based on 95 references [1,2,3,4,5,6,7,8,9,10,11,12,13,14,15,16,17,18,19,20,21,22,23,24,25,26,27,28,29,30,31,32,33,34,35,36,37,38,39,40,41,42,43,44,45,46,47,48,49,50,51,52,53,54,55,56,57,58,59,60,61,62,63,64,65,66,67,68,69,70,71,72,73,74,75,76,77,78,79,80,81,82,83,84,85,86,87,88,89,90,91,92,93,94,95]. We identified the utility of VR in neurosurgery, beyond planning, in the areas of neurosurgery training [2,8,9,10,11,12,13,14,15,16,17,18,19,20,21,22,23,24,25,26,27,28,29,30,31,32,33,34,35,36,37], neuronavigation [15,35,36,37,38,39,40,41,42,43,44,45,46,47,48,49,50,51], robotic neurosurgery [8,52,53,54,55], pain management [56,57,58,59,60,61,62,63,64,65,66], rehabilitation [67,68,69,70,71,72,73,74,75,76], and consent taking [77,78,79,80], as well as diagnostic tools [81,82,83,84,85,86,87,88,89,90,91,92,93,94,95] (Table 1).

## 4. Discussion

### 4.1. Brief History

Though VR was in use for panoramic viewing in the early eighteenth century, the first VR simulator was a flight simulator, invented in 1929, and the term “Virtual reality” was coined in 1987 [52]. The evolution of VR began with the induction of a young electrical engineer, Tom Furness of the United States Air Force [79]. This insightful invention resulted in him being labelled the “The Godfather of Virtual Reality” [57]. A VR system in medicine was first introduced by Robert Mann in orthopedics, followed by the induction of the head-mounted device (HMD) in the 1980s [60]. VR was first used in the treatment of arachnophobia (fear of spiders) in 1998, and this remains the first documented use of this technology in the treatment of pathology [64]. However, the first use of VR in the treatment of a neurosurgical disorder is relatively recent. This occurred in 2009 when David. B Clarke excised a Left frontal meningioma using the NeuroTouch neurosurgical simulator [80].

Throughout the literature, VR has been used interchangeably to denote VR, AR, and even mixed reality (MR). However, VR, AR, and MR are fundamentally different technologies. VR refers to computed generated three-dimensional (3D) immersive environments, AR refers to the projection of computer-generated images onto real-world images, and MR refers to the projection of virtual objects into the real world, where the objects are spatially aware and responsive [13]. Disadvantages of nausea, vomiting, temporarily impaired vision, and lack of sense of presence, noticed in the early usage, was due to the technical limitations of VR technology available at the time, as well as the inability of the human eye to fixate in-depth in a 3D-rendered image [13,39,87]. Similar disadvantages during surgery have also been reported with the use of high definition 4K 3D exoscopes [96,97]. This has been significantly addressed and improved with advances in technology and subsequent iterations of the concept.

### 4.2. Virtual Reality for Neuronavigation

AR and VR facilitate navigation during complex and otherwise difficult neurosurgical interventions, in addition to their other potential functions [44]. Two decades earlier, a report, published by neurosurgeons from Women and Brigham hospital, illustrated the potential advantage of using VR as a neuronavigation tool in 300 patients [41]. The report consisted of using VR by constructing a 3D model of the patient’s head and fusing this with real-time MRI on a “double doughnut-MRI” or open magnet MRI [41]. This facilitated the intraoperative navigation in minimally invasive surgeries by 3D construction of images preoperatively, an intraoperative fusion of these images with real-time MRI, and segmentation of the MRI, where essential landmarks and critical neurovascular structures were superimposed on the patient’s head on the screen [30]. In the words of Grimson et al. [41], “Virtual-reality technology is giving surgeons the equivalent of X-ray vision, helping them to remove tumors more effectively, to minimize surgical wounds and to avoid damaging critical tissues” [41]. Volume graph-guided neuronavigation highlights the role of AR as a navigation tool for complex neurosurgical interventions by converting the operating field into a virtual operating tool [15]. Microscope-assisted guided interventions (MAGI) provide 3D navigation, where preoperative 3D images are superimposed on the eyepieces of the operating microscope [39]. The advantage of MAGI, as compared to the pointer-based navigation system, is that the surgeon does not have to shift his focus from the operative site to the screen; additionally, MAGI provides information regarding the location of nearby structures, as compared to the structures visualized only under the pointer in a pointer-based navigation system [42]. Similarly, AR and VR are being used in image-enhanced endoscopy, where real and virtual images are superimposed with multiple layers to provide enhanced surgical exposure [45]. Bichlmeier et al. [38] introduced the concept of a virtual mirror in AR, similar to the utilization of the dental mirror, to visualize different aspects of the surgical territory. In a recent article, T. Fick et al. [40] have shown that a stand-alone AR HMD can be used in lieu of conventional neuronavigation, with significant advantages, such as direct superimposition of images the patient, improved ergonomics, and diminishing attention shifts [40]. The main benefit of AR HMD visualization in brain tumor surgery is the integrated continuous display, allowing for pointer-less navigation [46]. Navigation view provides the highest usability, while blocking the operative field less frequently. Roethe et al. [46] reported the use of the AR display in 44.4% of resection time, with a predominant AR type navigation view (75.7% of time), followed by target volume view (20.1% of time). Intraoperative VR has been described for critical mapping and discerning eloquent regions of the brain, especially in awake craniotomies. VR for awake craniotomy provides an immersive environment that is absent in traditional mapping techniques [47].

### 4.3. Virtual Reality as a Diagnostic Tool

Cerebrovascular surgery and neuro-interventional surgery rely heavily upon advanced neuroimaging techniques for operative decision-making and prognostication [83]. Clinical application of VR can augment the diagnostic accuracy and efficacy of these techniques [83]. Hybrid angio-suites enable neurointerventists to develop a VR immersive model, based on patient-specific anatomy for better procedural skill, training, and crisis resource management [84]. Unique metric-based performance assessment, outside the angio suite, and ability to perform complex neuro intervention, including mechanical thrombectomy, with the same set of principles as in a live patient, has been a great utility of VR technology [81]. Surgeons benefit from access to VR patient specific models too, for better diagnosing or planning management strategies [26,40], as well as for planning complex combined or hybrid procedures that require a combination of interventional and conventional surgical methods [73]. This advantage holds true, irrespective of the surgeons existing skill set and years of experience.

Another example of the clinical application of VR in diagnostics is to compare preoperative imaging with postoperative imaging, in order to identify junctional kyphosis in adult spinal deformity correction and eliminate its confounding effects on the sagittal alignment after the deformity correction procedure [17]. Taken together, this indicates that, although VR may not supplant traditional imaging modalities in the diagnosis of disorders, it allows for expansion of the fidelity and accuracy of these measures.

### 4.4. Virtual Reality in Neurosurgery Training

**Figure 1:**
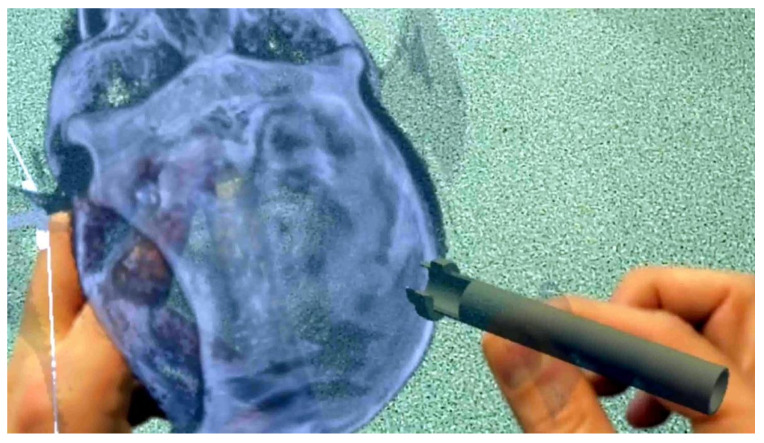
Application of VR for teaching placement of bed side ICP monitor via an external ventricular drain.

A detailed roadmap of neuroanatomy confers a higher degree of confidence and success in neurosurgical procedures [15]. This guiding principle has led to several advancements in the last few decades, addressing the needs of neurosurgical training and simulation (Figure 1) [2,15]. A key challenge faced by the neurosurgeon-in-training is performing a procedure with bimanual-dexterity in a narrow corridor bound by complex and vital neurovascular structures and non-resilient bones. Neuronavigation is heavily relied upon by neurosurgeons-in-training for planning their approach and localization; however, it is not suitable for developing spatial reasoning abilities and an over reliance on neuronavigation paradoxically extends the development of skills [1]. Moreover, as surgery progresses, the brain gradually shifts, which renders the navigation system, using preoperative images, less useful and accurate. To avoid this problem, an intraoperative brain imaging system (IBIS) was devised that identifies any discrepancy between intraoperative ultrasound and preoperative imaging [9]. Using IBIS, the intraoperative stimulation is modified in real-time, and inaccuracies are updated using AR [9,19]. VR, when used in simulation and training, proved to be a better alternative to reduce the cognitive load and operative stress duration, and proved to enhance efficacy for novice neurosurgeons [31]. VR tools available for neurosurgical education and training include a multifunction head-mounted display (HMD: Microsoft HoloLens, Google Glass), haptic feedback (NeuroVR, Immersive touch, Procedicus Vascular Interventional System Trainer (VIST)), synthetic tissue simulator (Creaplast, SynDaver, iDU optics 3D-printed models, Thomas Jefferson university durotomy repair module), VOSTARS (video and optical see-through augmented reality surgical systems) HMD-based surgical navigation platform, and surgical planning devices (surgical theatre, Dextroscope, VPI reveal, Synaptive Medical). VR and computer simulation are used in areas of medicine, military, and pilot training to reduce the danger(s) involved by providing a virtual simulator and visual and haptic feedback [12,17,71]. The challenge often faced is that physics-based simulators are computationally demanding and need resources, in terms of software and computing skills, to provide visual and haptic feedback, in addition to formal training in 3D immersive simulation [12,21,55]. HMD are the most engaging displays in VR, others being google glass, consisting of an LED or OLED display with a high refresh rate (120 Hz) and latency time of 20 milliseconds or less [18,28]. VR systems currently in use in neurosurgery training and simulation include part-task simulators (e.g., ventriculostomy catheter insertion models and subpial resection models), procedure simulation (NeuroTouch), surgical rehearsal platform (Dextroscope, virtual endoscopy), and robotic neurosurgery paradigms [8,20,35,84]. Further, VR plays an essential role in tele-proctoring, in order to train surgeons on complex procedures, regardless of their geographical location [11]. These immersive technologies impact global virtual connections, to empower low-and-middle-income countries and potential applications, in times of ongoing pandemics, such as COVID-19. Telemedicine will likely play an important role in future outpatient neurosurgery consultations, as this technology allows the surgeon to interact with the patient in a “merged reality” space, facilitating verbal, visual, and manual interaction between them [98,99,100]. Certain quality control standards should be adhered to while using VR technology as a neurosurgical educational tool. These include, but are not limited to, high-resolution images, appropriate sound quality, haptic and visual feedback, high processing power, internet speed, the structure of the organs, and tissue fidelity. Key advantages of using VR technology in neurosurgery training, as compared to human and animal models, are its low cost, non-invasive nature, limitless repetition ability, and the sheer variety and diversity in cases that can be simulated. However, realism and resolution are an ever-present concern [16]. The VR environment can be used to quantify surgeon performance, assess surgeon proficiency, and track training progression [101,102,103]. Young surgeons demonstrated greater enthusiasm and enjoyment in learning with AR HMD visualization [102]. Though more than 1000 articles were published on the use of VR in the medical sector, with significant numbers dedicated to neurosurgery, there are no controlled designed studies or high-quality studies with homogenous devices, thereby limiting the available evidence on the uses of VR technology in neurosurgery.

Research and technical developments in spinal surgery exceed other surgical specialties with advancements in navigation, endoscopy, and the advent of robotic spinal surgery. However, the development of AR, VR, and MR in spine and orthopedic surgery is similar to brain surgery, in terms of cost-effectiveness, levels of recommendations, evidence, and lack of standardized measures. Common spinal procedures, for which simulation-based training is currently used, in order of frequency, are pedicle screw insertion, vertebroplasty, posterior cervical laminectomy, and foraminotomy [10,23,24]. Leading simulator technologies in spinal surgery include Immersive Touch Simulator, Novint Falcon, Stealth 3D, and Osso VR, employing AR, VR, and MR techniques [10,22,23,30]. Gradually, the world has witnessed a parallel increase in literature on VR in minimally invasive spine surgery, spine endoscopy, tumor management, and spinal deformity correction [13,14,17,22,30,32].

### 4.5. Virtual Reality and Pain Management

In pain management, VR has initially been studied to treat trigeminal rhizotomy. Specifically, VR-assisted percutaneous radiofrequency trigeminal rhizotomy has shown safety and efficacy in studies and stresses the utility of virtual 3D CT scans in assessing the position and depth of the needle [56,58,61]. VR has also shown efficacy in managing pain during minor procedures and other chronic illnesses [60,62,63]. While this may not be strictly ‘neurosurgical‘, it is of importance in the ancillary management of patients during their stay in the neurosurgical service. Of particular importance in neurosurgery is that VR has shown to be efficacious in managing chronic pain that fails conventional opioid and physical therapy [59]. This opens avenues in the utility of VR for chronic pain disorders in neurosurgery, such as failed back and regional specific pain syndromes.

### 4.6. Virtual Reality in Rehabilitation

VR has been used in several novel ways, from creating a virtual environment to allow the patient to train in to the establishment of telerehabilitation services, in order to allow patients (who are understandably hard-pressed to travel) to pursue rehabilitation services at home, while their therapist can monitor progress from a different location [49,67,69,71]. In addition to this, in an interesting article by Christiansen et al. [67], VR was used to assess and quantify the level of cognitive deficit, following traumatic brain injury, by providing the patient with simple sequencing tasks. Nearly every rehabilitation domain has shown promise with the incorporation of VR, with examples including the assessment of driving with patients following trauma and providing leisure opportunities using video-based VR in young adults with physical and intellectual disabilities. Maresca et al. [72] reported a positive impact on cognitive domains, motor recovery, balance improvement, and reduction in anxiety and depression in a 60-year old individual with incomplete cervical spinal cord injury, treated with combined traditional physiotherapy and VR rehabilitation system. Another prospective study reported similar results on balance improvement in spinal cord injury patients treated with a VR rehabilitation system [73].

The advantages conferred by VR stem from the ability to gradually increase the complexity of tasks, facilitate simultaneous audio, visual, motor, and cognitive rehabilitation remain engaging and entertaining and offer positive, reward-based feedback, which are of paramount importance in preventing patient frustration and dropouts. Additionally, future strategies would be to make VR-based rehabilitation more universally accessible to promote patient compliance. When considering patients with long-term rehabilitative needs, VR and AR would be more economically viable, thereby alleviating the financial burden of an already distressed portion of society. In a seminal article, Weiss et al. [75] poignantly observes that there is real-world improvement in patients that correlate with improvement in the virtual world, and sufficient research and optimization should be directed towards improving VR rehabilitation systems.

### 4.7. Virtual Reality and Robotic Neurosurgery

The neuroArm leads this movement as an image-guided teleoperated neurosurgical robot [53]. As with all new technologies, a significant amount of learning and relearning must occur before the operator gains sufficient proficiency. This creates a unique problem, as surgeons find themselves requiring training, in a short period of time (as dramatically new interventions have a latent lead time, followed by an exponential growth phase), with restricted access to the instrumentation being studied [8]. The utility of VR, in allowing surgeons to train in the absence of readily deployed systems or time slots with those systems, has precedence in the field of laparoscopy [93]. A more recent example is the pattern of adoption faced with the da Vinci Surgical System (Intuitive Surgical, Sunnyvale, California) and its companion VR trainer, the DV-Trainer (Mimic Technologies, Seattle, Washington) [54]. The same may be anticipated as robotic neurosurgery becomes more widely adopted. VR, in this interesting application, traces its roots back to its earliest widespread adoption, as similar to the field of avionics, ‘trainers’ can be developed designed on the same master console or interface used by the surgical robot [8]. Added advantages are that, by designing the system on the same interface, the incorrect transfer of motor skills and haptic mismatch does not occur, as the development of a haptic interface that mimics an instrument is a difficult task [70]. VR systems are additionally more cost-effective than additional robotic units, which require installation for training. Moreover, although there is a surge in robotic neurosurgery in different neurosurgical pathologies, most robotic neurosurgery advancement is limited to the computer interface between the surgeon and patient, further validating the use of VR trainers [52].

An exciting vision of the future of robotics integrated with AR was presented by Madhavan et al. [52], where a telerobotic system was fused with an AR overlay that allows for the dexterity and precision of the robotic system, fused with the added information (such as projected position(s) of vital structures and the pathology in question) from an AR overlay. Added functionality to the system can also be brought in using AR to measure distances and implants, obviating the need for conventional measuring devices and consolidating instrument diversity (which is especially advantageous in a robotic system).

### 4.8. Consent Taking and VR

Informed consent (IC) is defined as “the communication process between a patient and physician that results in the patient’s authorization or agreement to undergo a specific medical intervention”. Neurosurgery is a high-risk specialty, with the highest rates of legal claims among all other specialties [77]. In a recent randomized controlled trial [78], 33 patients were randomized 1:1:1 to three groups. In the two experimental groups, patients underwent 3D, immersive, informed consent with two different surgical planners (group 1 and group 2). In the control group, patients underwent an informed consent, supported by traditional 2D radiological images. All these patients were asked to fill the Spielberger state and trait anxiety self-evaluation questionnaire (state anxiety inventory (STAI Y-1) and trait anxiety inventory (STAI Y-2)), to assess individual situational and trait anxiety (higher scores reflect higher anxiety levels) after standard surgical consent and the planner(s) explained the surgical aspects and nuances using the Surgical Theater™ and Vesalius™ surgical planning platforms [65]. The patients in the VR groups appreciated this communication experience, while their objective comprehension was higher (score mean (SD): group 1 82.65 (6.83); group 2 77.76 (10.19)), as compared with the control group (57.70 (12.49); *p* < 0.001) [61]. Although, the idea may be controversial, as providing extra information about surgical risks and complications may augment patients’ anxiety, especially when complications can be disabling. However, this avenue of VR utilization goes beyond regular neurosurgical planning and needs to be investigated significantly to reduce medicolegal risks and ensure patient understanding.

### 4.9. Additional Avenues and Challenges of VR in Neurosurgery

VR has been utilized for training non-technical skills, such as communication, teamwork, and situational awareness in health care professionals [34]. Extrapolating on this idea, VR and AR may, in the future, additionally find applicability in the training of surgical scrub nurses and operating room technicians, who require significant exposure before attaining the technical proficiency required of them. Although stated in the context of robotic assistants, the words of Laligam Shekar et al. state that “when observed, such surgeon–assistant teams appear to be as graceful as a ballet or symphony. But such a team takes time and active effort to develop” [89]. The answer to this might lie in the field of VR. VR has also shown promise in mapping social cognition during awake craniotomy and other complex cognitive functions that are routinely not mapped during surgery [66,92]. Katsevman et al. [47] reported the use of a VR protocol as a feasible functional tool in awake-patient brain tumor surgery by using it as a complement during cognitive screening, in addition to language testing. Similarly, Mazerand et al. [50] showed the use of intraoperative visual field assessment with a virtual reality headset during direct subcortical stimulation, to map the optical radiations and prevent a permanent visual field defect during awake surgery (a promising approach).

Significant technical hurdles faced during awake surgery, such as mapping of the optic radiation, have also been overcome with the utility of VR [50].

Finally, VR has also been used to improve patient compliance and comfort. Studies have shown the utility of VR in preparing patients for MRI examinations and VR headsets are commonly used to prevent the intense discomfort faced by patients while undergoing the MRI [9,68]. These examples further indicate that the utility of VR and AR, which was initially limited by technological sophistication, is now inherently limited by artificially narrowed horizons.

VR has faced challenges, predominantly due to the technical complexities in designing clinically valuable and relevant models. The main issues that have halted the monumental rise of VR in neurosurgery include, but are not limited to, feasibility in application and transference to real-life scenarios, ethics in supplementing with standard practices, and cost-effectiveness analyses. One of the critical drawbacks of VR application in neurosurgery, beyond presurgical planning, is the learning curve associated with it, when used to augment surgical procedures; this is much more significantly evident with the advancement of robotic neurosurgery [52,86]. This steep learning curve in VR-assisted neurosurgery is an additional burden to the already time-consuming neurosurgical residency, which often requires further training and fellowships to acquire competence and expertise in specific neurosurgical procedures [16].

### 4.10. Strengths and Limitations of the Study

The evaluation of neurosurgical performance, in which VR is used, is a newborn field of interest. This review has the feature of showing the different fields in which AR and VR are used in neurosurgery, as well as their possible implications in the next future. However, it has the limitations that, in order to limit the broad topic dealt and not disperse the information gathered in this review, tractography and white matter integrity in neuronavigation were not discussed.

## 5. Conclusions

VR and AR show benefits in preoperative planning and multimodal neuronavigation for spine and brain surgery. In addition, the included studies suggested that VR and AR have beneficial effects for medical education and neurosurgical training. This paper reported the immense possibilities of VR in neurosurgery, beyond merely planning for surgical procedures. To generate relevant evidence in the next future, we need to rigorously evaluate AR and VR implementations, in order to better understand the strengths and limitations of HMD and other tools used during surgery, as well as in all fields of neurosurgery. The importance of VR and AR, especially in “social distancing”, in neurosurgery training for economically disadvantaged sections, prevention of medicolegal claims, and pain management and rehabilitation is promising and warrants further research.

## Figures and Tables

**Table 1:** Studies exploring the use of VR, beyond neurosurgical planning, as well as their advantages and disadvantages.

Issues Addressed for the Use of VR As:	Studies	Advantages	Disadvantages
Neurosurgery training	Abhari et al. [2]; Chan et al. [8]; Drouin et al. [9]; Gasco et al. [10]; Higginbotham et al. [11]; Hooten et al. [12]; Hu et al. [13]; Hu et al. [14]; Kockro et al. [15]; Konakondla et al. [16]; Lafage et al. [17]; Lee et al. [18]; Lee et al. [19]; Lemole et al. [20]; Lobel et al. [21]; Lohre et al. [22]; Moult et al. [23]; Pfandler et al. [24]; G. Riva et al. [25]; Montemurro et al. [26]; Robison et al. [27]; Sabbadin et al. [28]; Sabbagh [29]; Wei et al. [30]; Weigl et al. [31]; Wewel et al. [32].	World-wide applicability	Real world situations can be radically more challenging
		Cost-effective	False sense of confidence
		Multiple scenarios practiced	
		Stimulation of stress situations	
2. Neuronavigation	Bichlmeier et al. [38]; Edwards et al. [39]; Fick et al. [40]; Grimson et al. [41]; King et al. [42]; Kockro et al. [15]; Condino et al. [43]; Kockro et al. [44]; Shahidi et al. [45]; Roethe et al. [46]	Margins of resection improved	Brain shift may lead to errors
		When visual differentiation is lost, enables identification	Falling over of pial margins prevents ideal use
		Critical margins can be pre-sought	
3. Robotic neurosurgery	Chan et al. [8]; Madhavan et al. [52]; Pandya et al. [53]; Lee et al. [54]; Ramaswamy et al. [55]	Increased precision	Real world feedback absent
		Critical areas: tremor, suturing	Unsupervised errors possible
		More degree of freedom of movement	
4. Pain management	Christiano et al. [56]; Meng et al. [58]; Pourmand et al. [59]; Bani Mohammad et al. [60]; Shakur et al. [61]; Walker et al. [62]; Wong et al. [63].	Reduces medication dependence	False sense of relief of pain
		Prevents depressive symptoms	
5. Rehabilitation	Christiansen et al. [67]; Davies et al. [69]; Gourlay et al. [49]; Davies et al. [71]; Maresca et al. [72]; Sengupta et al. [73]; Schultheis et al. [74]; Weiss et al. [75]; Weiss et al. [76].	Allows effective training	Real world situations can be more challenging
		Cost-effective	
		Wide applicability	
6. Consent taking	Jena et al. [77]; Perin et al. [78].	Allows patients to better understand procedure	Actual procedure may be different hence chance of false security
		“Informed” in the real sense	
7. Diagnostic tool	Lafage et al. [17]; Liebig et al. [81]; Mitha et al. [83]; Rudarakanchana et al. [84]; Incekara et al. [86]; Sekhar et al. [89]; Roh et al. [91]; Delion et al. [92]; Steineke et al. [94].	Stimulate various situations	May lead to false diagnosis
		Added tool	
